# Causal Algebras on Chain Event Graphs with Informed Missingness for System Failure

**DOI:** 10.3390/e23101308

**Published:** 2021-10-06

**Authors:** Xuewen Yu, Jim Q. Smith

**Affiliations:** 1 Statistics Department, University of Warwick, Coventry CV4 7AL, UK; j.q.smith@warwick.ac.uk; 2 The Alan Turing Institute, London NW1 2DB, UK

**Keywords:** Chain Event Graphs, interventions, causal calculus

## Abstract

Graph-based causal inference has recently been successfully applied to explore system reliability and to predict failures in order to improve systems. One popular causal analysis following Pearl and Spirtes et al. to study causal relationships embedded in a system is to use a Bayesian network (BN). However, certain causal constructions that are particularly pertinent to the study of reliability are difficult to express fully through a BN. Our recent work demonstrated the flexibility of using a Chain Event Graph (CEG) instead to capture causal reasoning embedded within engineers’ reports. We demonstrated that an event tree rather than a BN could provide an alternative framework that could capture most of the causal concepts needed within this domain. In particular, a causal calculus for a specific type of intervention, called a remedial intervention, was devised on this tree-like graph. In this paper, we extend the use of this framework to show that not only remedial maintenance interventions but also interventions associated with routine maintenance can be well-defined using this alternative class of graphical model. We also show that the complexity in making inference about the potential relationships between causes and failures in a missing data situation in the domain of system reliability can be elegantly addressed using this new methodology. Causal modelling using a CEG is illustrated through examples drawn from the study of reliability of an energy distribution network.

## 1. Introduction

The use of Bayesian Networks (BN) for the study of reliability has been widely advocated in the literature [1]. However, the asymmetric processes that are common in system reliability can hardly be fully captured by the framework of a BN.

Fortunately, it has been shown that any discrete BN can be embellished into a tree-based graph called a Chain Event Graph (CEG) [2,3]. The CEG is a graphical model that is a function of an underlying event tree and certain context specific conditional independence statements. In particular, the CEG can model and depict the types of structural asymmetries that the BN framework struggles to embody [4]. This can then provide a framework for studying the causal mechanisms behind the failures of a given system. For example, Cowell and Smith [2] developed a dynamic programming algorithm for maximum a posterior (MAP) structural learning for causal discovery within a restricted class of CEGs called stratified CEGs.

Conventional causal algebras have been adapted from Pearl’s *do*-calculus for BNs [5] to the singular manipulation on a CEG, and the *back-door* theorem has been generalised to estimate the effect of this manipulation by previous research [6,7]. In a different strand of research, Barclay, Hutton, and Smith [8] developed a class of CEGs suited for incorporating various missing data structures directly through its topology. Unlike BNs, conjugate inference is still well supported by the structure of CEGs even in the presence of missingness [2].

In Section 2, we adapt the MAP structural learning algorithm [2] to search for the best scoring structure of a CEG when some data is informedly missing. The selected model provides the best explanation of the observed data that has been informedly censored. By assuming that each candidate CEG is causal in the sense formally defined in [6,7], the best scoring CEG is of a CEG in idle mode, and then causal deductions can be made from it.

In our recent work [9], we demonstrated how to embed the causal reasoning underlying engineering reports for CEGs designed specifically for applications in system reliability. The causal calculus we developed there only provided a framework to study and analyse the impact of *remedial interventions*, i.e., interventions designed to rectify the root cause after a failure had been observed.

In Section 3, we extend the use of the CEG causal framework with missingness to express and analyse a different kind of intervention called a *routine intervention*. This new class of intervention is necessary when we are evaluating the impact of interventions within scheduled maintenance regimes. These regimes are prepared in advance and are used to inspect machines with the objective of preventing future failures that might be about to happen. In this context, although the data may be informedly missing, we can still develop algorithms that, under certain stated hypotheses, produce formulae to give quantitative estimates of the impacts of various candidate routine interventions of this type.

In this paper, we can, therefore, show how we can use the underlying CEG model to predict the effect of various types of such interventions. In particular we report a new back-door criterion—an analogue of Pearl’s back-door criterion for BNs [5]. This gives a quick sufficient condition as to whether the effect of such an intervention is identifiable when data is censored in a way that induces informed missingness. This criterion significantly increases the scope of the original causal calculus using CEGs designed for the singular manipulation [6] and the stochastic manipulation established for BNs [5]. It, thus, enables us to transfer causal technologies so that they apply to this graphical family.

In Section 4, we demonstrate how to interpret the causal structures of a best scoring CEG by a simple example of a conservator system. Furthermore, comparative experiments are designed to show that the proposed new causal algebras can embellish the current structural learning algorithm to capture the causal effects of a routine intervention.

The contributions of this paper are threefold. First, we formally derive a method for selecting a CEG providing the framework of a probability model of maintenance regimes, which acknowledges the presence of informed missingness within the fitted data endemic in these applications. Second, we devise new causal algebras for the routine intervention and prove the identifiability of its causal effects in presence of the types of missing data that we might expect from this application. Third, we demonstrate how important this new intervention calculus can be in making valid inferences and how naive inferences that treat the system as uncontrolled and ignore the underlying causal structure within this application can severely mislead the analyst.

## 2. Causal Identifiability on Chain Event Graphs with Informed Missingness

We begin this section by briefly reviewing and then extending the definition of a CEG [2,3,6,7,8] before providing a systematic approach to embedding information about the context-specific missingness into a CEG customised for the domain of reliability [9,10].

Suppose we have a vector of variables $X=(X_{1},X_{2}...,X_{n})$ taking values in a state space $X=X_{1}\times \cdots \times X_{n}$, among which we explore various putative causal hypotheses. An *event tree*$T\left(X\right)=\left(V_{T},E_{T}\right)$ can be constructed to represent relationships embedded in X, where $V_{T}$ denotes the vertex set and $E_{T}$ denotes the edge set of T(X). Each non-leaf node is also called a *situation*. Let $S_{T}$ denote the set of non-leaf nodes. The *floret* of a situation $v\in S_{T}$ is a subtree of T(X), denoted by $F\left(v\right)=\left(V_{F(v)},E_{F(v)}\right)$. The vertex set of F(v) consists of *v* and the vertices in $S_{T}$ connected from *v* by a directed edge in $E_{T}$: $V_{F(v)}=\left\{v\right\}\cup \left\{v^{\prime }\in V_{T}|e_{v,v^{\prime }}\in E_{T}\right\}$. The edge set of F(v) is a subset of $E_{T}$ satisfying $E_{F(v)}=\left\{e_{v,v^{\prime }}:v^{\prime }\in V_{T},e_{v,v^{\prime }}\in E_{T}\right\}$.

Let $F_{T}={\left\{F\left(v\right)\right\}}_{v\in S_{T}}$ denote the collection of all florets on the tree T. Let $\mu (v_{0},v)$ denote a subpath from the root node $v_{0}$ to a node $v\in V_{T}$ on the event tree. Every floret F(v) represents a random variable conditional on $\mu (v_{0},v)$. We denote this conditional random variable by $X\left(v\right)=X|\mu \left(v_{0},v\right)$ for X∈X. Each emanating edge $e_{v,v^{\prime }}$ of *v* is labelled by a value x∈X(v). Thus, every conditional variable $X_{i}$, i∈{1,...,n}, is represented on a set of florets on the event tree, denoted by $F(v\left(X_{i}\right))$. Previous research [3,4,6,7] has demonstrated the capability of a tree-like structure to encode the asymmetric information. The corresponding event tree T associated with this description can be asymmetric and non-stratified [2,4] so that the florets representing the same variable can have different distances from the root node $v_{0}$.

**Figure 1:**
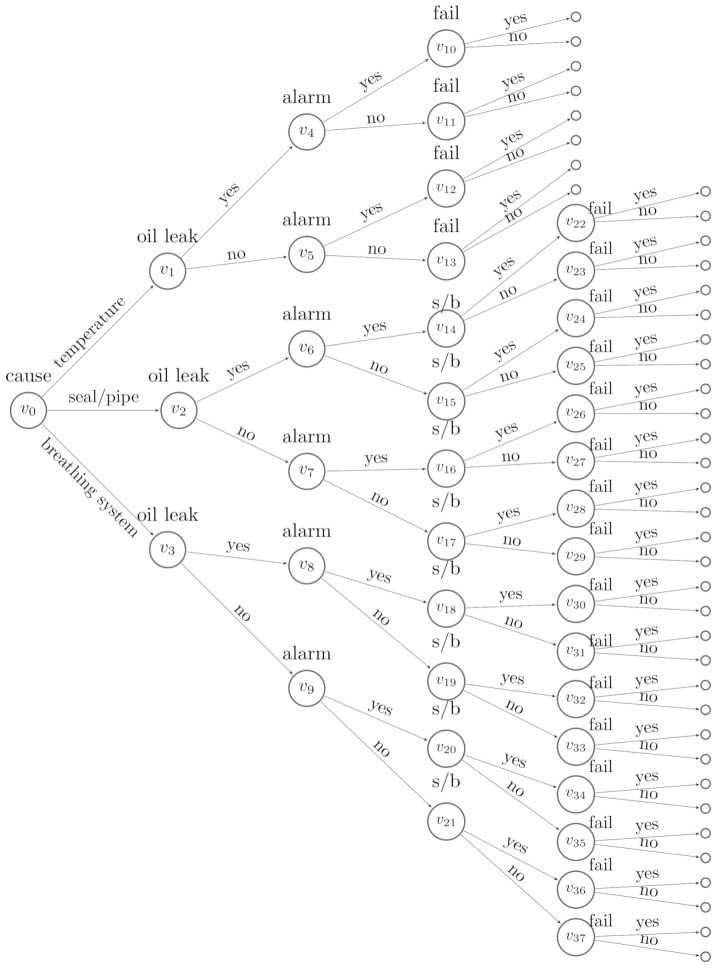
An event tree constructed for the conservator system of a transformer.

Figure 1 depicts an event tree for a conservator system. Its variables are $X=(X_{cause},$$X_{leak},X_{alarm},X_{s/b},X_{fail})$. The categorical variable $X_{cause}$ represents causes of defects and has three levels {temperature,seal/pipe,andbreathingsystem}; $X_{leak}$ is the oil leak indicator; $X_{alarm}$ is the alarm indicator; $X_{s/b}$ is an indicator of whether there is a sight glass defect or a buchholz defect; and $X_{fail}$ is a failure indicator. This tree is constructed under the assumption that the fault caused by low temperature is irrelevant to the sight glass or buchholz defect, labelled as s/b on the tree. The situations of the tree are annotated as $\left\{v_{0},\cdots ,v_{37}\right\}$, and the leaves are the unlabelled vertices. Since the last variable modelled on this tree is $X_{fail}$, the leaves represent the status of the conservator being failed or operational.

Let $\Lambda_{T}$ denote the set of all root-to-leaf paths on the tree and $\lambda \left(v,v^{\prime }\right)\in \Lambda_{T}$ denote the root-to-leaf paths passing through vertices $v,v^{\prime }\in V_{T}$. The vector $\theta_{v}=P\left(X\left(v\right)\right)=P\left(X|\mu \left(v_{0},v\right)\right)$ is called the vector of primitive probabilities. Let $\theta_{T}={\left(\theta_{v}\right)}_{v\in V_{T}}$, which satisfies $\sum_{v^{\prime }\in ch\left(v\right)}\theta_{v,v^{\prime }}=1$ and $\theta_{v,v^{\prime }}\in \left(0,1\right)$ for all $v\in V_{T}$, where $ch\left(v\right)=\left\{v^{\prime }\in V_{T}|e_{v,v^{\prime }}\in E_{T}\right\}$. Then, the pair $\left(T\left(X\right),\theta_{T}\right)$ indexes the *probability tree*[2,3] defined over X.

The BN is capable of handling the missing data whenever this applies to all values of a pre-assigned set of variables by assigning a missingness indicator to each unobservable variable within that set. It is, therefore, possible to use the BN as a framework for identifying when causal hypotheses are identifiable in this rather restricted setting. The associated analyses use various graphically stated criteria—such as the front-door and the back-door criteria—see e.g., [11,12,13]. However, unfortunately, the types of missingness that routinely occur in reliability—and, in particular, those associated with the data we collect when performing routine maintenance—are rarely missing across the original random vector associated with the system in this sort of symmetric way. This is because we only learn about those parts of a system that we have chosen to inspect.

In contrast, the probability tree provides a natural and more flexible way to visualise and model the context-specific missingness, where the unobservability of the variable partially depends on which path it lies on the tree. Here, we import the informed missingness into the event tree by defining the *floret-dependent missingness* [14]. Thus, consider a floret F(v), if the value of the corresponding variable X(v) is unobservable, then we classify this floret into $F\left(v\right)\in F^{M}$.

On the other hand, if conditioned on $\mu (v_{0},v)$, the value of the variable X(v) is always observed, and then the corresponding floret is classified into $F\left(v\right)\in F^{O}$. Accordingly, we have two subsets of florets, $F^{M}$ and $F^{O}$, representing unobservable florets and fully observed florets, respectively. Then, $F^{M}\cap F^{O}=\emptyset$ and $F^{M}\cup F^{O}=F_{T}$. For every unobservable floret $F\left(v_{j}\right)\in F^{M}$, we define a *missing floret indicator* as:

(1) $$B_{F(v_{j})}=\left\{\begin{array}{cc} 1\; & \mathrm{if}\;x(v_{j})\;\mathrm{is}\;\mathrm{missing},\\ 0 & \mathrm{otherwise}. \end{array}\right.$$

Then, $B_{F(v_{j})}$ represents the conditional missingness and

(2) $$P\left(B_{F(v_{j})}=1\right)=P\left(X\left(v_{j}\right)\;\mathrm{missing}|\mu \left(v_{0},v_{j}\right)\right).$$

**Figure 2:**
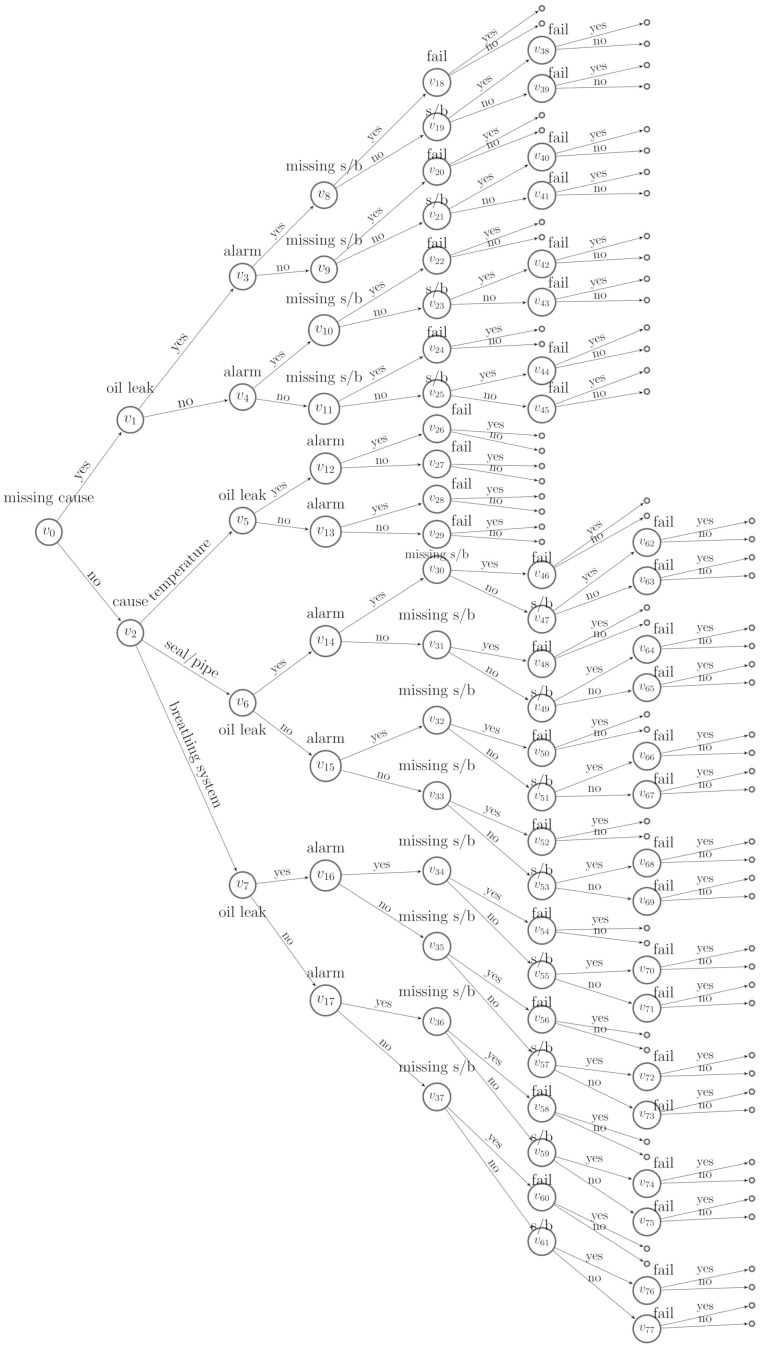
A missingness event tree constructed from Figure 1.

When $P\left(B_{F(v_{j})}=1\right)\in \left(0,1\right)$, we construct a floret representing this indicator, denote this by $F(v\left(B_{F(v_{j})}\right))$, and call it a *missing indicator floret*. We then reconstruct an event tree by importing the missing indicator florets on to T. We call this a *missingness event tree* (m-tree). Here, we assume that $B_{F(v_{j})}$ precedes $X(v_{j})$, denoted by $B_{F(v_{j})}≺X\left(v_{j}\right)$. In particular $F(v_{j})$ is appended to the edge emanating from $v(B_{F(v_{j})})$ labelled by $B_{F(v_{j})}=0$. This artificially introduced ordering has already been shown to be useful for interpreting an event tree constructed with informed missingness [8]. The m-tree then has a new class of florets $F^{MI}=F\left(v\left(B\right)\right)$ for $B={\left\{B_{F}\right\}}_{F\in F^{M}}$, which is the set of missing indicator florets. The variables associated to the m-tree are expanded to (X,B). We denote the topology of the m-tree by T(X,B). An example of the missingness event tree is shown in Figure 2.

Having a missingness event tree, we further elicit a *missingness staged tree* from T(X,B). For two situations *v* and *w*, if F(v) and F(w) represent the same variable, then these two situations are in the same stage whenever $\theta_{v}=\theta_{w}$[3], and the emanating edge $e_{v,v^{\prime }}$ is labelled the same value of *X* as $e_{w,w^{\prime }}$ when $\theta_{v,v^{\prime }}=\theta_{w,w^{\prime }}$. Here, we relax the restrictions for a *stratified staged tree* where two situations in the same stage have the same distance from the root node [2,4]. For example, $v_{18}$ can be in the same stage as $v_{38}$ in the missingness event tree in Figure 2, similar example see [8].

**Figure 3:**
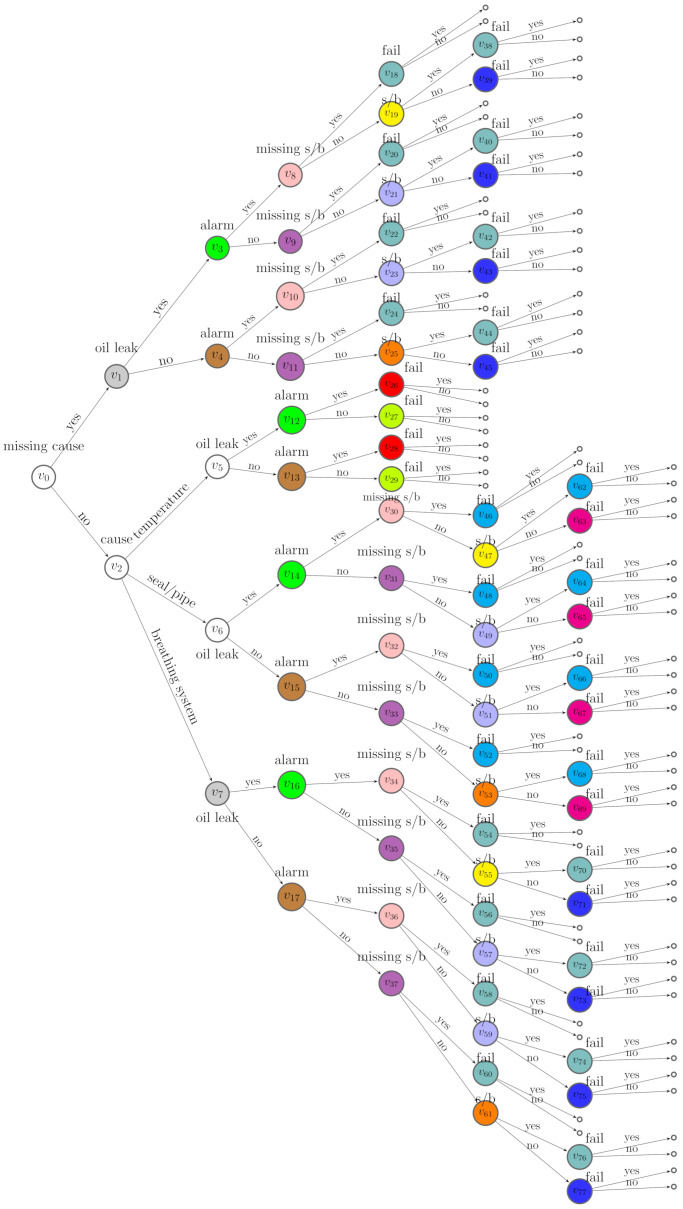
A missingness staged tree of the m-tree in Figure 2.

Here, we assume that situations along the same root-to-leaf path cannot be in the same stage. This is the *square-free* condition defined by Collazo et al. [3]. Vertices in the same stage are assigned a unique colour, and the edges emanating from the same stage with the same label are assigned the same colour. Such a coloured tree that embeds context-specific conditional independence relations is a missingness staged tree. Let $U=\{u_{1},..,u_{l}\}$ denote the set of stages in the m-tree. Let $u(X_{i})$ represent the set of stages associated with variable $X_{i}$ and $U\left(X\right)=\{u\left(X_{1}\right),\cdots ,u\left(X_{n}\right)\}$. Let U(B)=U/U(X) denote the set of stages associated to the missing floret indicators. An example of a missingness staged tree of the m-tree in Figure 2 is depicted in Figure 3.

Two situations $v_{j},v_{k}\in u_{i}\in U$ in the same stage are in the same *positionw* if the rooted subtrees $T_{v_{j}}\left(X,B\right)$ and $T_{v_{k}}\left(X,B\right)$ are isomorphic. This clustering gives a finer partition of vertices than *U*, denoted by $W=\{w_{1},...,w_{m}\}$. A *missingness chain event graph* (MCEG) $C\left(X,B\right)=\left(V_{C},E_{C}\right)$ can be constructed from a missingness staged tree as follows. A sink node $w_{\infty }$ is created by merging all the leaves of T(X,B). Then, the vertex set is $V_{C}=W⋃w_{\infty }$.

For any two $w,w^{\prime }\in V_{C}$, we create an edge for every v∈w and the child node $v^{\prime }\in ch\left(v\right)\in V_{T}$, which belongs to the position $w^{\prime }$, where the annotating edge probability is the same as that of $e_{v,v^{\prime }}\in E_{T}$ and is inherited from the original tree. The colours of the vertices and edges of the MCEG are the same as the corresponding stages and edges in the missingness staged tree [15].

Note that the events on the event tree are chronologically ordered. By definition, a cause comes before its effects. We can be reasonably confident in providing X with a plausible order. For example, the trajectory of the events that lead to a machine’s failure always starts with a cause, followed by symptoms, and terminates with a failure. Therefore, we can construct event trees for analysing system failures following this order. In this case, having failed or not is always modelled on the leaves of the tree. Examples are shown in Figure 1 and Figure 2.

It follows that, for this special application of CEGs in system reliability, it is convenient to adapt the semantics and to replace the sink node $w_{\infty }$ defined above by $w_{\infty }^{f}$ and $w_{\infty }^{n}$. In this way, $w_{\infty }^{f}$ is the receiving node of the edges labelled by a failure, while $w_{\infty }^{n}$ is the receiving node of the edges labelled by an operational condition.

**Figure 4:**
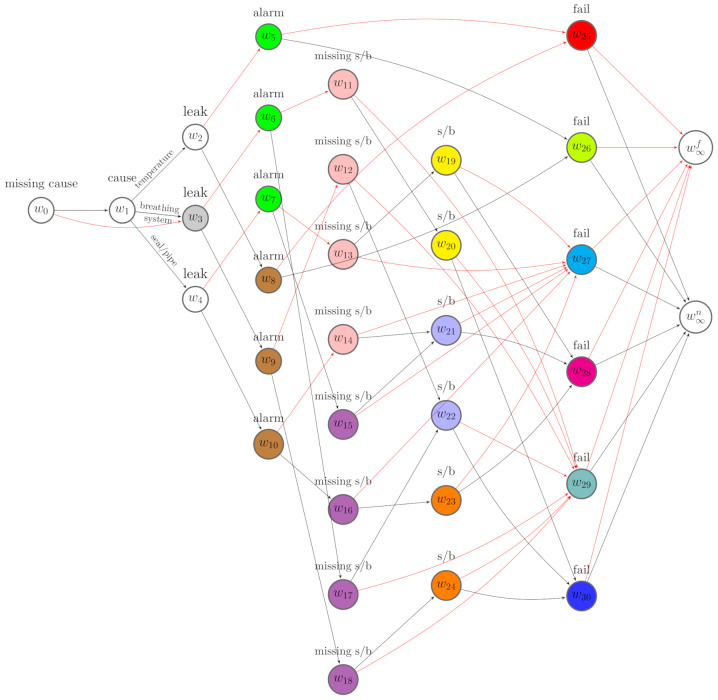
A MCEG derived from Figure 3. For simplicity, the edges labelled “no” are coloured in red.

Thus, we can classify the root-to-sink paths into two categories: failure paths and deteriorating paths. The former terminate in $w_{\infty }^{f}$, while the latter terminate in $w_{\infty }^{n}$. Figure 4 gives an example of such a MCEG derived from Figure 3.

It is possible to perform conjugate inference on an idle MCEG even when the data is informed censored [8,16]. This enables us to greatly speed up the search for good explanatory models. The simplest prior to set up in this context assumes each stage vector $\theta_{u}=\left(\theta_{u_{1}},...,\theta_{u_{l}}\right)$ is independently Dirichlet with parameters $\left(\alpha_{u1},...,\alpha_{um_{u}}\right)$ [3,8]. This is identical to the case when there are no missingness indicators:

(3) $$f\left(\theta |C\left(X,B\right)\right)=\prod_{u\in U}\frac{\Gamma (\sum_{j=1}^{m_{u}}\alpha_{uj})}{\prod_{j=1}^{m_{u}}\Gamma \left(\alpha_{uj}\right)}\prod_{j=1}^{m_{u}}\theta_{uj}^{\alpha_{uj}}.$$

Let $\alpha_{u}=\sum_{j=1}^{m_{u}}\alpha_{uj}$ so that, in particular, the equivalent sample size is $\alpha =\sum_{u\in U}\sum_{j=1}^{m_{u}}\alpha_{uj}$.

Then, given a set of observations *D*, the posterior can be computed in a closed form due to Dirichlet-multinomial conjugacy. Thus,

(4) $$\begin{array}{cc} f(\theta |D,C(X,B)) & =\prod_{u\in U}f\left(\theta_{u}|D,C\left(X,B\right)\right)\\ \; & =\prod_{u\in U}\frac{\Gamma (\sum_{j=1}^{m_{u}}\alpha_{uj+})}{\prod_{j=1}^{m_{u}}\Gamma \left(\alpha_{uj+}\right)}\prod_{j=1}^{m_{u}}\theta_{uj}^{\alpha_{uj+}}, \end{array}$$

where $\alpha_{uj+}=\alpha_{uj}+n_{uj}$, and $\alpha_{u+}=\alpha_{u}+n_{u}$ is the updated parameter vector.

The log-likelihood score for a MCEG C(X,B) can be decomposed into local scores associated with the variables X and the missingness indicators B.

(5) $$\log Q\left(C\left(X,B\right)\right)=\log f_{C(X,B)}\left(D\right)=\sum_{i=1}^{n}\log Q_{u(X_{i})}\left(C\left(X,B\right)\right)+\sum_{u\in U(B)}\log Q_{u}\left(C\left(X,B\right)\right)$$

We can explicitly compute the log-likelihood in a closed form:

(6) $$\log Q\left(C\left(X,B\right)\right)=\sum_{u\in U}\left(\log\Gamma \left(\alpha_{u}\right)-\log\Gamma \left(\alpha_{u+}\right)-\sum_{j=1}^{m_{u}}\left(\log\Gamma \left(\alpha_{uj}\right)-\log\Gamma \left(\alpha_{uj+}\right)\right)\right).$$

To elicit a best scoring CEG from an event tree, it is necessary to search over all possible orderings over the variables modelled by the tree when the total order over the variables is unknown. The event tree is defined to be built with respect to X, and the associated missingness event tree is built as a function of T(X) with appropriate hypotheses of missingness. Therefore, even when the dataset has missing values, we still only search over permutations over X to find an appropriate ordering that best explains the observed process.

Let Π denote an ordering of X. This could be a set of partial orderings. All variables represented on the m-tree can automatically be ordered given Π. We denote the m-tree with a specified ordering Π by $T\left(X\left(\Pi \right),B\right)=\left(V_{T},E_{T}\right)$.

It is non-trivial to identify causal structure from a finite observational dataset. However, the idle model first needs to be estimated before any causal relations can be explored. Many advances have been made in casting the causal discovery as a Bayesian model selection problem [2,17,18]. The MAP structural learning algorithm is a popular and well-developed tool for selecting a best topology of CEGs that best explains the data.

Under the hypothesis that there are no unobserved confounders [2], we render the best scoring structure selected by the MAP algorithm causal and assume it is the model of the idle system when there is no intervention imported. This enables us to further perform causal analysis. Given such a causal graph, we can derive causal hypotheses from the structure and estimate causal effects under different hypothesised underlying causal mechanisms.

Sometimes there is only a putative partial order rather than total order on the variables X whose causal relationship needs to be explored. However, in this setting we can still perform the search over candidate CEGs for the best fitting model, providing that the missing variables only extend to later nodes of the tree.

Cowell and Smith [2] and Collazo et al. [3] presented a recursive algorithm to find the best sink variable for every subset of X ordered by increasing size. This algorithm can be simply adapted for the tree built for the informedly missing data. Let $X_{j}\subseteq X$ denote the subset of variables whose ordering is needed to be learned and $\Pi_{X_{j}}$ denote the best partial ordering over $X_{j}$.

Then, through applying the algorithm designed by [2,3] on every $X_{j}$, we can find the best ordering over the variables defined on the tree, where $\Pi ={\left\{\Pi_{X_{j}}\right\}}_{j}$. Here, we search over subspaces $X_{j_{1}}\times \cdots \times X_{j_{k}}$ for $X_{j_{1}},...,X_{j_{k}}\in X_{j}$ and compute the local scores with respect to the corresponding Y. In particular, for every $X_{j}^{\left(l\right)}=\left\{X_{j_{1}},...,X_{j_{l}}\right\}$, where l∈{1,...,k−1}, we find a best sink variable $X^{\prime }\in X_{j}^{\left(l\right)}$ for every $X_{j}^{\left(k-1\right)}\subseteq X_{j}^{\left(k\right)}$ that has been ordered appropriately. The best sink variable $X^{\prime }$ is found by computing the local score of the best subtree spanned by $X_{j}^{\left(k-1\right)}⋃\left\{X_{s}\right\}$ for every $X_{s}\in X_{j}^{\left(k\right)}$ together with the corresponding missingness indicators.

The MAP score can be evaluated directly from the local scores that have been computed because the total score is the sum of local scores as shown in Equation (5). Two MCEGs $C_{1}$ and $C_{2}$ with respect to the same data set can be compared by the log-posterior Bayes factor. Suppose both trees have Dirichlet priors whose hyperparameters are $\alpha_{1}$ and $\alpha_{2}$. The Bayes factor, then, has a closed form [3]:

(7) $$lpBF\left(C_{1},C_{2}\right)=\log q\left(C_{1}\right)-\log q\left(C_{2}\right)+\log Q\left(C_{1}\right)-\log Q\left(C_{2}\right),$$

where $\log q(C_{i})$ denotes the log prior. Different priors over models can be chosen given expert judgement on different missingness mechanisms and conditional dependencies. When using a uniform prior, $\log q\left(C_{1}\right)-\log q\left(C_{2}\right)=\frac{1}{N_{C}}-\frac{1}{N_{C}}=0$, where $N_{C}$ denotes the total number of models.

## 3. Causal Algebras for Routine Maintenance

By assuming the best scoring CEG causal and treating it as the idle system, one can always design experiments to collect data under the influence of interventions, and thus we can estimate the causal effects from the partially observed system. By controlling certain events on the tree, the semantics of a causal CEG allow us to explore its effect on the events that lie downstream of the controlled events along the root-to-sink paths. For a reliability analysis, it is extremely useful to trace and discover the potential causes of abnormal conditions or failures. By designing causal algebras for different interventions, we can make predictive inferences about the effects of a variety of types of maintenance and thus improve the prediction of system failures.

Having defined the remedial intervention on the CEG for the reliability system in [9], here, we investigate a new class of intervention regime. In the reliability literature, there are two main categories of maintenance: *corrective maintenance* (CM) and *preventive maintenance* (PM) [19]. CM takes place after a failure, while PM often refers to a scheduled maintenance that helps to identify and prevent problems during inspections before a failure occurs [20]. In this section, we carefully customise causal algebras for the intervention in light of the latter case, calling this a *routine intervention*. A routine intervention not only has an impact on the lifetime of the maintained equipment but also affects the likelihood of different defects that may occur in the equipment.

### 3.1. Effects on Lifetime

In the context of reliability, the interventions largely consist of replacing failed components of the system. This type of intervention—unusual in most causal analyses—requires special attention, especially as there are some very well-known effects of such interventions that need to be incorporated before it is possible to realistically model the effects of interventions. In particular, when describing the failure of equipment, the bathtub effect [20] is widely applicable. This divides the lifetime of an equipment into three periods: the early life of a new component has a decreasing failure rate; this is followed by a period with a constant failure rate; the failure rate rises during the wear-out period [21]. A Weibull distribution whose density is given by

(8) $$f\left(t\right)=\frac{\beta }{\eta }{\left(\frac{t}{\eta }\right)}^{\beta -1}e^{-{\left(\frac{t}{\eta }\right)}^{\beta }}$$

is often used by reliability engineers to model this varying hazard [20], where the scale parameter is η>0, and the shape parameter is β>0. The survival function takes the form:

(9) $$1-F\left(t\right)=e^{-{\left(\frac{t}{\eta }\right)}^{\beta }}.$$

Let $\Lambda_{C}$ denote the set of all root-to-sink paths on the MCEG C(X(Π),B). Then, the lifetime of the repaired equipment can be modelled on the associated root-to-sink paths, denoted by $\tilde{\Lambda }\subseteq \Lambda_{C}$. For $\lambda \in \tilde{\Lambda }$, let T(λ) represent the total lifetime of the equipment when the failure trajectory is modelled on the path λ.

For a repairable system, the PM prolongs the life of the component [22,23,24]. By adopting the Arithmetic Reduction of Age (ARA) model, which assumes the life of the equipment is shortened up to proportionality [23], we now establish methods to evaluate the effect of the scheduled PM on the equipment’s lifetime.

Let $Z_{s}^{\lambda }$ represent the failure time of an equipment with observed age *s* given a failure process that is modelled on the path λ. Then, the survival function is

(10) $$P\left(Z_{s}^{\lambda }>t\right)=\frac{1-F_{\lambda }\left(s+t\right)}{1-F_{\lambda }\left(s\right)}=e^{-{\left(\frac{s+t}{\eta_{\lambda }}\right)}^{\beta_{\lambda }}+{\left(\frac{s}{\eta_{\lambda }}\right)}^{\beta_{\lambda }}},$$

where $F_{\lambda }\left(\;\cdot \;\right)$ denotes the reliability distribution for failure trajectory λ.

In an idle system, for $\lambda \in \tilde{\Lambda }$, T(λ) has the same distribution as $Z_{0}^{\lambda }$, i.e., $T\left(\lambda \right)\overset{d}{=}Z_{0}^{\lambda }$. Thus,

(11) $$P\left(T\left(\lambda \right)>t\right)=P\left(Z_{0}^{\lambda }>t\right)=1-F_{\lambda }\left(t\right).$$

Preventive maintenance can be scheduled periodically. However, for simplicity, we only demonstrate the effect of a single time routine maintenance in this paper. We suppose that an equipment is diagnosed during a routine maintenance and is repaired at age τ. Kijima [24] and Guessoum and Aupiedy [23] introduced a parameter representing the degree of repair, denoted by A∈[0,1]. When A=0, the repair is *perfect* and restores the maintained part to *as good as new* (AGAN). On the other hand, A=1 corresponds to a *minimal repair*, after which the maintained part is functioning as it was just prior to the repair.

Since the repaired equipment is rejuvenated, the virtual age [23,24] after maintenance is then Aτ. Let $T^{*}\left(\lambda \right)$ denote the post-intervention time to failure. Then, after a routine intervention, the residual lifetime of the maintained equipment has the same distribution as $Z_{A\tau }^{\lambda }$. Therefore,

(12) $$P\left(T^{*}\left(\lambda \right)>t\right)=P\left(Z_{A\tau }^{\lambda }>t\right)=\frac{1-F_{\lambda }\left(t+A\tau \right)}{1-F_{\lambda }\left(A\tau \right)}.$$

### 3.2. Manipulations on the MCEG

If $X_{i}\in X$ takes value $x_{ij}$, let $e\left(x_{ij}\right)\in E_{C}$ denote the edges labelled by this value that emanate from $w(X_{i})$. The set of vertices receiving $e(x_{ij})$ are represented by $w(x_{ij})$. The path related probability, denoted by π(λ) for $\lambda \in \Lambda_{C}$, can then be factorised as:

(13) $$\pi \left(\lambda \right)=\prod_{e\in E_{\lambda }}\theta_{e},$$

where $E_{\lambda }$ represents a collection of edges lying along the path λ.

When there is a routine intervention, we are only interested in the process portrayed by the deteriorating paths. We denote this set of paths by $\Lambda_{x_{fail,0}}=\Lambda \left(e\left(x_{fail,0}\right)\right)$, where $x_{fail,0}$ represents $X_{fail}=0$. Whatever this preventive action is, an analogue of the *do*-operation $do(X_{fail}=0)$ is imported into the idle MCEG. Thus, we force $e(x_{fail,0})$ to have probability 1 and $e(x_{fail,1})$ to have probability 0, or, equivalently, we manipulate $\Lambda_{x_{fail,0}}$. Therefore, we always have the post-intervened path probability:

(14) $${\hat{\pi }}^{\Lambda_{x_{fail,0}}}\left(\lambda \right)=\left\{\begin{array}{cc} \frac{\prod_{e\in E_{\lambda }}\theta_{e}}{\theta_{e(x_{fail,0})}} & \mathrm{if}\;\lambda \in \Lambda_{x_{fail,0}};\\ 0 & \mathrm{otherwise}, \end{array}\right.$$

This is a singular manipulation on the MCEG and yields a manipulated MCEG with respect to $\Lambda_{x_{fail,0}}$. We denote this by ${\hat{C}}^{\Lambda_{x_{fail,0}}}$.

Depending on the preventive action taken, other manipulations can also be imported into the MCEG in addition to the singular manipulation on $\Lambda_{x_{fail,0}}$. We next demonstrate two scenarios of composite manipulations.

#### 3.2.1. Composite Singular Manipulations under Routine Intervention

In this section, we discuss the situation where the preventive maintenance perfectly repaired a problem, and, as a consequence of this repair, an event $x_{r}$ is forced to occur. The event $x_{r}$ is labelled on a set of edges $e(x_{r})$ whose receiving nodes are $w(x_{r})$ and emanating nodes are $pa(w\left(x_{r}\right))$. In this case, the unit will be forced to pass through every edge $e\in e(x_{r})$ with probability 1. We, therefore, have a composition of singular manipulations, and the manipulated events are $x=\{x_{fail,0},x_{r}\}$. On an MCEG, the controlled event is represented by

(15) $$\Lambda_{x}=\Lambda \left(e\left(x\right)\right)=\Lambda \left(e\left(x_{fail,0}\right)\right)\cap \Lambda \left(e\left(x_{r}\right)\right).$$

Let F(e(x)) denote the set of florets that the edges e(x) lie in.

If we are interested in the effect of the routine maintenance on event *y*, then, on the MCEG, we represent it by $\Lambda_{y}=\Lambda \left(e\left(y\right)\right)$. The set of florets that e(y) lies in is denoted by F(e(y)).

Given a CEG, let $\pi (\Lambda_{y}||\Lambda_{x})$ denote the probability of observing event *y* given a manipulation that forces the events x to occur. We aim to estimate this probability from the observed data and to demonstrate that the effects of a routine intervention are identifiable. We have shown in [9] that causal effects from a singular manipulation are estimable, also called *recoverable*, by adapting the back-door theorem [5]. Here, we simply extend our previous results [9] so that it now also applies to the types of composite manipulations that we discuss here.

The MCEG provides flexible choices of events z to be the back-door partition so that $\Lambda_{z}$ partitions $\Lambda_{C}$[6,7]. We first impose a constraint on z that $F\left(e\left(z\right)\right)⊈F^{MI}$, i.e., that cannot be a missingness indicator. This is to ensure that $\pi (\Lambda_{y}||\Lambda_{x})$ can be estimated from the partially observed data [9]. Note that any of F(e(x)),F(e(y)),F(e(z)) might be unobservable. Let

(16) $$F_{x\cup y\cup z}=\left\{F:F\in F\left(e\left(x\right)\right)\cup F\left(e\left(y\right)\right)\cup F\left(e\left(z\right)\right)\;\mathrm{and}\;F\notin F^{MI}\right\}.$$

We define the *manifest paths* to be the largest set of root-to-sink paths on the MCEG passing along edges labelled by x, *y* and z. We let $b_{F(e(x)),0}={\left\{b_{F,0}\right\}}_{F\in F(e(x))}$ denote the set of missingness indicators of florets F(e(x)) taking value 0, i.e., values of the corresponding floret variables are observed. Then, the manifest paths are

(17) $$\Lambda \left(w\left(b_{F_{x\cup y\cup z,0}}\right)\right)=\Lambda \left(w\left(b_{F(e(x)),0}\right)\right)\cap \Lambda \left(w\left(b_{F(e(y)),0}\right)\right)\cap \Lambda \left(w\left(b_{F(e(z)),0}\right)\right).$$

We can construct a sub-MCEG $C^{M}$ using the manifest paths. Let the collection of the root-to-sink paths of this subgraph be $\Lambda_{C^{M}}=\Lambda \left(w\left(B_{F_{x\cup y\cup z,0}}\right)\right)$. We call this sub-MCEG the *manifest MCEG*. This construction ensures that there is no edge in the manifest MCEG associated with a controlled event, effect, or partition event being missing.

We next reconstruct $\pi (\Lambda_{y}||\Lambda_{x})$ from the manifest MCEG. Let $\pi^{\Lambda_{C^{M}}}(\Lambda_{y}\left|\right|\Lambda_{x})$ denote the probability of observing an event *y* given a manipulation forcing x to happen within the manifest MCEG. Note that the manipulated MCEG is a subgraph of the manifest MCEG. For a singular manipulation on $\Lambda_{x}$, the manipulated paths on the manifest MCEG are

(18) $$\Lambda_{*}=\Lambda \left(w\left(b_{F_{x\cup y\cup z,0}}\right)\right)\cap \Lambda_{x}.$$

The manipulated MCEG with respect to $\Lambda_{*}$ is then denoted by ${\hat{C}}^{\Lambda_{*}}$ and satisfies $\Lambda_{{\hat{C}}^{\Lambda_{*}}}=\Lambda_{*}$.

**Theorem** **1** (The m-back-door criterion for composite singular manipulations). *When a dataset has missing values, the effect of a singular manipulation on x on y is identifiable on the MCEG if we can find a partition $\Lambda_{z}$ of $\Lambda_{C^{M}}$ such that*

(19) $$\pi^{\Lambda_{C^{M}}}(\Lambda_{y}\left|\right|\Lambda_{x})=\sum_{z}\pi \left(\Lambda_{y}|\Lambda_{x},\Lambda_{z},\Lambda \left(w\left(b_{F_{x\cup y\cup z,0}}\right)\right)\right)\pi \left(\Lambda_{z}|\Lambda \left(w\left(b_{F_{x\cup y\cup z,0}}\right)\right)\right).$$

For the proof of this theorem, see [9,14].

**Example** **1.** *Given the causal MCEG in Figure 4 of a conservator system, we demonstrate how the formulae defined above works for a specific routine maintenance that successfully prevents an oil leak. This is equivalent to importing a combination of $do(X_{fail}=0)$ and $do(X_{leak}=0)$ operations to the idle MCEG. The controlled events are $x=\{x_{fail,0},x_{leak,0}\}$. From Figure 4, we next identify the associated root-to-sink paths. In particular,*

(20) $$\Lambda_{x_{fail,0}}=\underset{w\in \{w_{25},\cdots ,w_{30}\}}{⋃}\Lambda \left(e_{w,w_{\infty }^{n}}\right),$$

(21) $$\Lambda_{x_{leak,0}}=\Lambda \left(e_{w_{2},w_{8}}\right)\cup \Lambda \left(e_{w_{3},w_{9}}\right)\cup \Lambda \left(e_{w_{4},w_{10}}\right),$$

*and $\Lambda_{x_{fail,0},x_{leak,0}}=\Lambda_{x_{fail,0}}\cap \Lambda_{x_{leak,0}}$.*

*To next focus on alarm, the effect event is $x_{alarm,1}$. The associated set of paths is $\Lambda_{x_{alarm,1}}=$$\Lambda (e_{w_{5},w_{25}})\cup$$\Lambda \left(e_{w_{6},w_{11}}\right)\cup \Lambda \left(e_{w_{7},w_{13}}\right)\cup \Lambda \left(e_{w_{8},w_{25}}\right)\cup \Lambda \left(e_{w_{9},w_{12}}\right)\cup \Lambda \left(e_{w_{10},w_{14}}\right)$. The causal query with respect to x is identifiable whenever $\pi (\Lambda_{x_{alarm,1}}||\Lambda_{x_{fail,0},x_{leak,0}})$ can be recovered from the MCEG by estimating it from the dataset with missing entries. There are a variety of possible choices for the partition events z. Here, we simply let z be $X_{cause}$ whose corresponding positions lie upstream of the controlled events $x_{leak,0}$ on the tree. The corresponding floret is, then, F(e(z))$=F(w_{1})$.*

*We now construct the manifest MCEG and the manipulated MCEG in order to identify the effects of the intervention. Notice that the controlled events and the effect events are always observable in our example. Thus,*

(22) $$\Lambda \left(w\left(b_{F(e(x)),0}\right)\right)=\left(\underset{w\in \{w_{25},\cdots ,w_{30}\}}{⋃}\Lambda \left(w\right)\right)\cap \left(\underset{w\in \{w_{2},w_{3},w_{4}\}}{⋃}\Lambda \left(w\right)\right)=\Lambda_{C},$$

(23) $$\Lambda \left(w\left(b_{F(e(y)),0}\right)\right)=\underset{w\in \{w_{5},\cdots ,w_{10}\}}{⋃}=\Lambda_{C},$$

*However, the back-door partition events might be missing. The collection of paths along which z are observed is*

(24) $$\Lambda \left(w\left(b_{F(e(z)),0}\right)\right)=\Lambda \left(w_{1}\right).$$

Following Equation (17), the manifest paths are $\Lambda \left(w\left(b_{F_{x\cup y\cup z,0}}\right)\right)=\Lambda_{C}\cap \Lambda \left(w_{1}\right)=\Lambda \left(w_{1}\right)$. Thus, to investigate this, we construct the manifest MCEG with respect to $\Lambda (w_{1})$. This is a subgraph of the idle MCEG in Figure 4 obtained by simply removing the edge $e_{w_{0},w_{3}}$, which represents the causes that are missing. We further elicit the manipulated MCEG from the manifest MCEG. By the definition of the manipulated paths given in Equation (18), we select the manipulated paths from the manifest paths: $\Lambda_{*}=\Lambda \left(w_{1}\right)\cap \Lambda_{x_{fail,0},x_{leak,0}}$. Since the intervention forces $x_{fail,0}$ and $x_{leak,0}$ to happen, the events $x_{fail,1}$ and $x_{leak,1}$ should never be observed. Thus, the probability of a manipulated path passing along the edges $e(x_{fail,1})$ and $e(x_{leak,1})$ is 0.

**Figure 5:**
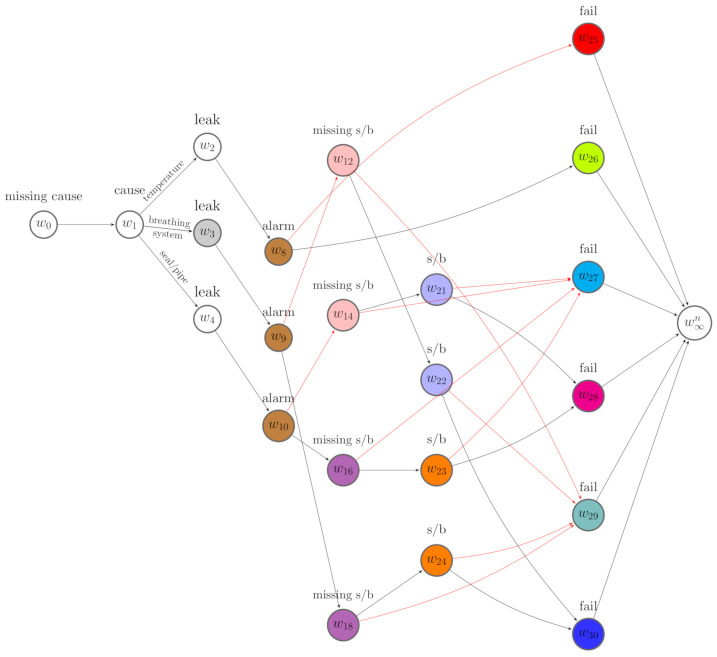
The manipulated MCEG when controlling $x_{leak,0}$ and $x_{fail,0}$

Equivalently, the positions $w\left(x_{fail,1}\right)=w_{\infty }^{f}$ and $w\left(e\left(x_{leak,1}\right)\right)=\left\{w_{5},w_{6},w_{7}\right\}$ should never be passed through by any path in the manipulated graph. Then, by removing the nodes and edges that are not traversed by the manipulated paths in the manifest MCEG, we can derive the manipulated MCEG with respect to $\Lambda_{*}$, see Figure 5. We can then estimate the causal effects on alarm using the formula given in the m-back-door theorem defined above. The conditional path probabilities in Equation (19) can simply be evaluated using the factorisation of the corresponding primitive probabilities in the manipulated MCEG.

#### 3.2.2. Composite Singular and Stochastic Manipulations under Routine Intervention

During routine inspections, the field engineers may clean the components, check the oil level and leakage, replace some units, and so on [25]. Since there are different types of repair and because the degree of this repair varies, the manipulations enacted by the routine intervention could be more complicated than forcing a specific event to happen. In fact, repairing or replacing an equipment could affect multiple units or multiple defects of a unit.

Therefore, depending on the repaired subcomponent and the degree of repair, multiple florets can be influenced separately and simultaneously. Thus, a routine intervention could introduce more uncertainty to the probability distributions over these relevant florets. Therefore, the distributions of some of the primitive probabilities may need to be reassigned. This manipulation is then called a stochastic manipulation on the MCEG.

Unlike a remedial intervention [9], a stochastic manipulation induced by a routine intervention is not restricted to root causes. Consider a floret F whose distribution is manipulated by a routine intervention. The events represented by this floret could be defects or symptoms of the maintained equipment.

Let $x_{r}$ denote the controlled events of a routine intervention. Suppose we can find the edges labelled by these events, denoted by $e(x_{r})$, then $F(e\left(x_{r}\right))$ is the set of florets whose distribution are manipulated under the routine intervention. Let $w^{*}=pa\left(w\left(x_{r}\right)\right)$ denote the set of emanating nodes of edges $e(x_{r})$. We can then conclude that $F\left(w^{*}\right)=F\left(e\left(x_{r}\right)\right)$.

For $w\in w^{*}$, we update the probability distribution after a routine intervention via the transformation:

(25) $$\hat{q}\left(\theta_{w}\right)=G\left[q\left(\theta_{w}\right)\right]$$

where $\hat{q}\left(\;\cdot \;\right)$ represents the post-intervened distribution. The transformation *G* preserves the properties of the transition probabilities so that $\sum_{e\in E(w)}\theta_{e}=1$ and $\theta_{e}>0$.

Motivated by the steady model [26,27], one straightforward option is to map distributions to distributions through non-linear state space models. A possible transformation to increase uncertainty in a distribution is the *power steady transformation* [26,28], which can be characterised by information loss after the intervention takes.

(26) $$\hat{q}\left(\theta_{w}\right)\propto q{\left(\theta_{w}\right)}^{\phi },$$

where ϕ∈(0,1]. Assume that the value of ϕ can be assessed and informed by the domain experts. Then, a power steady evolution assumes that such information loss is linear and proportional to ϕ so that:

(27) $$E\left[\log\hat{q}\left(\theta_{w}\right)\right]=\phi E\left[\log q\left(\theta_{w}\right)\right]+c,$$

for some constant *c*.

For a Dirichlet prior $\theta_{w}∼Dirichlet\left(\alpha_{w}\right)$ with concentration parameters $\alpha_{w}=\left(\alpha_{w1},\cdots ,\alpha_{wm_{w}}\right)$, following [29], we can transform it to $Dirichlet({\hat{\alpha }}_{w})$, where ${\hat{\alpha }}_{w}=\left({\hat{\alpha }}_{w1},\cdots ,{\hat{\alpha }}_{wm_{w}}\right)$ and ${\hat{\alpha }}_{wj}-1=\phi \left(\alpha_{wj}-1\right)$, for $j\in \{1,\cdots ,m_{w}\}$. By this transformation, the mode remains the same. We can consider such manipulations when searching for the best scoring MCEG for causal discovery. This is explained in Section 4.

Having updated the transition probabilities, the path probabilities under the stochastic manipulation given a routine intervention can be re-evaluated. Let $\Lambda (w^{*})$ denote the set of root-to-sink paths on the MCEG passing through any position $w\in w^{*}$. Let $\bar{\Lambda }\left(w^{*}\right)=\Lambda_{C}/\Lambda \left(w^{*}\right)$. Then, the probabilities of paths in $\Lambda_{x_{fail,0}}\cap \Lambda \left(w^{*}\right)$ are affected by both the singular manipulation on $x_{fail,0}$ and the stochastic manipulation on $F(w^{*})$. The probabilities of paths in $\Lambda_{x_{fail,0}}\cap \bar{\Lambda }\left(w^{*}\right)$ are affected by the singluar manipulation on $x_{fail,0}$. Therefore, the post-intervened path probabilities on the MCEG are:

(28) $$\hat{\pi }\left(\lambda \right)=\left\{\begin{array}{cc} \frac{\prod_{e\in E_{\lambda }}\theta_{e}}{\theta_{e(x_{fail,0})}\prod_{e^{\prime }\in E\left(w^{*}\right)\cap E_{\lambda }}\theta_{e^{\prime }}}\times \prod_{e^{\prime }\in E\left(w^{*}\right)\cap E_{\lambda }}{\hat{\theta }}_{e^{\prime }} & \mathrm{if}\;\lambda \in \Lambda_{x_{fail,0}}\cap \Lambda \left(w^{*}\right),\\ \frac{\prod_{e\in E_{\lambda }}\theta_{e}}{\theta_{e(x_{f,0})}} & \mathrm{if}\;\lambda \in \Lambda_{x_{fail,0}}\cap \bar{\Lambda }\left(w^{*}\right),\\ 0 & \mathrm{otherwise}. \end{array}\right.$$

Let $x^{*}$ denote the set of all events represented on $F(w^{*})$ and let $x=x_{fail,0}\cap x^{*}$ denote the set of events that are manipulated. Then, the set of florets associated with the manipulated events, the effect event and the partition events is

(29) $$F_{x\cup y\cup z}=\left\{F:F\in F\left(e\left(x_{fail,0}\right)\right)\cup F\left(w^{*}\right)\cup F\left(e\left(y\right)\right)\cup F\left(e\left(z\right)\right)\;\mathrm{and}\;F\notin F^{MI}\right\}.$$

The manifest paths are defined analogously to Equation (17) so that no event of interest, i.e., x,y,andz, is missing in this restricted class of paths.

(30) $$\Lambda \left(w\left(B_{F_{x\cup y\cup z,0}}\right)\right)=\Lambda \left(w\left(b_{F(e\left(x_{fail,0}\right)),0}\right)\right)\cap \Lambda \left(w^{*}\right)\cap \Lambda \left(w\left(b_{F(e(y)),0}\right)\right)\cap \Lambda \left(w\left(b_{F(e(z)),0}\right)\right).$$

We next show the identifiability of the effects by adapting the back-door criterion for stochastic manipulation [9]. More specifically, this is possible whenever we need to identify a $\Lambda_{z}$ that partitions the root-to-sink paths of the manifest MCEG $C^{M}$ so that

(31) $$\begin{array}{cc} \pi^{\Lambda_{C^{M}}}(\Lambda_{y}\left|\right|\Lambda_{x_{fail,0}},{\hat{\theta }}_{w^{*}})=\sum_{x\in x}\sum_{z} & \pi \left(\Lambda_{y}|\Lambda_{x},\Lambda_{z},\Lambda \left(w\left(b_{F_{x\cup y\cup z},0}\right)\right)\right)\pi \left(\Lambda_{z}|\Lambda_{x},\Lambda \left(w\left(b_{F_{x\cup y\cup z},0}\right)\right)\right)\\ \; & \times \hat{\pi }\left(\Lambda_{x}|\Lambda \left(w\left(b_{F_{x\cup y\cup z},0}\right)\right)\right), \end{array}$$

where

(32) $$\hat{\pi }\left(\Lambda_{x}|\Lambda \left(w\left(b_{F_{x\cup y\cup z},0}\right)\right)\right)=\frac{\hat{\pi }\left(\Lambda_{x},\Lambda \left(w\left(b_{F_{x\cup y\cup z}}\right)\right)\right)}{\hat{\pi }\left(\Lambda \left(w\left(b_{F_{x\cup y\cup z},0}\right)\right)\right)}.$$

The numerator and denominator are the post-intervened path probabilities. Note that these can be computed using Equation (28). Assuming that a stochastic manipulation on ${\hat{\theta }}_{w^{*}}$ is equivalent to forcing each *x* with probability $\pi (\Lambda_{x}||{\hat{\theta }}_{w^{*}})$ for every $x\in x^{*}$ [5], we can obtain Equation (31) by expressing the causal query as

(33) $$\pi^{\Lambda_{C^{M}}}(\Lambda_{y}\left|\right|\Lambda_{x_{fail,0}},{\hat{\theta }}_{w^{*}})=\sum_{x\in x^{*}}\pi^{\Lambda_{C^{M}}}(\Lambda_{y}\left|\right|\Lambda_{x_{fail,0},x})\pi^{\Lambda_{C^{M}}}\left(\Lambda_{x}|\Lambda_{x_{fail,0}},{\hat{\theta }}_{w^{*}}\right).$$

The first component on the right hand side of the equation can be evaluated by applying the results in Equation (19), and the second component can be simplified to Equation (32). By doing this, we have the expression in Equation (31).

**Example** **2.** Given the idle system in Figure 4, suppose routine maintenance involved in checking the oil level, cleaning the leakage, and topping up the oil, but this did not fully prevent the oil leak. The manipulations imported to the idle system under this intervention are then different from the one we discussed in Example 1. Suppose florets $F\left(w_{2}\right),F\left(w_{3}\right),F\left(w_{4}\right)$ are directly affected in response to the maintenance. Then, these florets are stochastically manipulated, and $w^{*}=\left\{w_{2},w_{3},w_{4}\right\}$. This gives the same $\Lambda (w\left(b_{F(e(x)),0}\right))$ as in Example 1. If we are interested in how the sight glass or buchholz defect is affected by this intervention, then the effect event is $x_{s/b,1}$. Note that this event is unobservable and $\Lambda \left(w\left(b_{F(e\left(x_{s/b,1}\right)),0}\right)\right)={⋃}_{w\in \{w_{19},\cdots ,w_{24}\}}\Lambda \left(w\right)$.

**Figure 6:**
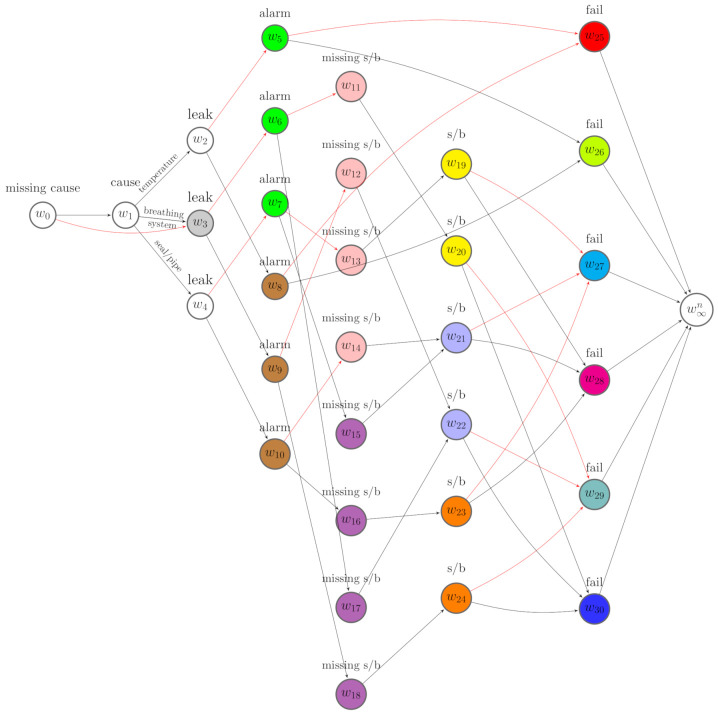
The manipulated MCEG for Example 2.

Here, we can choose $X_{alarm}$ as the partition events z, and these are always observable. Next the manifest MCEG is constructed from the idle MCEG by removing the paths that do not traverse any position in $\left\{w_{19},\cdots ,w_{24}\right\}$. The manipulated MCEG is obtained by further deleting the paths that terminate in $w_{\infty }^{f}$ from the manifest MCEG, see Figure 6. If the post-intervention probabilities ${\hat{\theta }}_{w^{*}}$ are known, then we can evaluate the path probabilities in the manipulated MCEG following the factorisations we specified in Equation (28). Then, conditional on the manifest paths, each probability in Equation (31) can be computed to estimate the effects of the observed maintenance on the sight glass or the buchholz.

## 4. Experiments

Due to commercial sensitivity, we cannot disclose the real maintenance data from the energy distribution company and examine our methodology on it. Here, we show experimentally, using synthetic data, how the structural learning algorithm over a class of MCEGs can be used to provide useful causal inferences. We, then, perform a comparative study to demonstrate how the predictions are improved when incorporating the causal algebras we specified in previous section into the algorithm for the synthetic experimental data.

### 4.1. Causal Discovery with the Structural Learning Algorithm

Assume a ground truth missingness staged tree in Figure 3 and a corresponding MCEG in Figure 4 are valid. Assume the causal ordering here is $\Pi_{1}=X_{cause}≺X_{leak}$$≺X_{alarm}≺X_{s/b}≺X_{fail}$. The oil leak, alarm, and sight glass or buchholz defect are faults that may appear before a failure or routine maintenance. Thus, the oil leak could be a potential cause of alarm and the defect in buchholz or sight glass. We assume that, for any floret, the parameters of primitive probability vector are independent, and the vectors of primitive probabilities associated with each stage are mutually independent.

This ensures a model search based on product of independent Dirichlet priors over the model parameters and a closed-form conjugate analysis [30]. Based on these assumptions, we now generate observation data $D_{1}$ of size 5000 from the ground truth MCEG with the corresponding hypothesized transition probabilities. This emulates the dataset in a situation when there has been no intervention to the system.

To begin to learn a best model for $D_{1}$ given the event tree in Figure 2, we specify the Dirichlet hyperparameters. We use established methods and treat each $\alpha_{uj}$ as the number of phantom units [3], which is believed to arrive at $j^{th}$ child of stage *u*. We let the total phantom units entering the root vertex $v_{0}$ be 1 and denote this by α=1.

**Figure 7:**
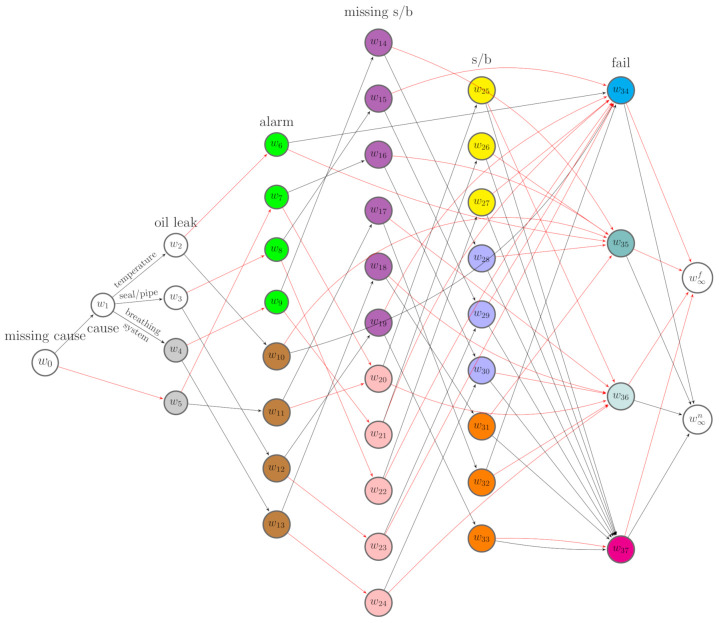
The best scoring MCEG selected for $D_{1}$ with hypothesised causal ordering $\Pi_{1}$.

By performing the MAP algorithm, the best scoring MCEG is shown in Figure 7. In this MCEG, denoted by $C(X\left(\Pi_{1}\right),B)$, the positions representing the same variable $X_{i}\in X$ are vertically aligned in descending order with respect to $P(X_{i}\left(w\right)=1|D_{1},\mu \left(w_{0},w\right))$. For transparency, the edges that are supposed to have a label “yes” have been coloured red for clarity.

The posterior means for each stage are summarised in Table 1. The score of this selected model is −20,389.83. The stages for $X_{leak}$, $X_{alarm}$ and the missing indicator of s/b defect in this tree are accurately learned by the algorithm when these are compared with the stages in the ground truth MCEG. In terms of the stages for $X_{s/b}$, the stage assigned to $v_{23}$ is wrong. There are 15 misclassifications appearing for $X_{fail}$. One possible reason is that the dataset is not sufficiently large to provide sufficient information on the last event modelled on the tree.

The best scoring MCEG in Figure 7 has a complex topology because many stages for the last variable modelled on the tree are misspecified. However, we can still summarise some causal explanations from it when assuming it is causal. We read the causal relationships from the semantics of a causal CEG in an analogous way to a causal BN [3,6]. For example, from Figure 7, we see that all the edges representing oil leak point to the stage $u^{\prime }=\left\{w_{6},\cdots ,w_{9}\right\}$, which is coloured in green, while the edges representing no leak point to the stage $u^{\prime \prime }=\left\{w_{10},\cdots ,w_{13}\right\}$, which is coloured in brown. The stage $u^{\prime }$ is located above $u^{\prime \prime }$ on the tree, meaning the mean posterior probability of alarm at this stage is higher than that at $u^{\prime \prime }$.

Therefore, the oil leak gives rise to the likelihood of alarm. Root causes also lie upstream of alarm on the tree and can affect the possibility of alarm. However, from Figure 7, whether the cause is missing and which cause is observed appear to have no influence on alarm given an oil leak. Thus, given the oil leak, the alarm is independent of the root causes we specified for this model. We could say that the oil leak is the main cause of alarm given the hypothesised causal ordering $\Pi_{1}$. One causal implication of this discovery is that we could prevent an alarm by fixing or preventing the oil leak. For positions associated with failure indicators, $w_{37}$ is aligned at the lowest position. This means that the probability $P(X_{fail}=1|\mu \left(w_{0},w_{37}\right),D_{1})$ is the lowest compared with the probability of failure conditional on the position $w_{34}$ or $w_{35}$ or $w_{36}$. There are eight edges pointing to $w_{37}$ labelled by no s/b defect and only one edge pointing to it labelled by a s/b defect. Thus, to increase the reliability of the machine, we can schedule the preventive maintenance for the sight glass or the buchholz.

### 4.2. A Comparison Study

Now, we assume the routine intervention described in Example 2 has occurred, and Figure 4 portrays the real causal structure. We, then, simulate synthetic data $D_{2}$ of size 5000 from this intervened model to emulate an experimental dataset by the following setups. First, we assume the 5000 pieces of equipment here have been intervened in the same way by the same routine maintenance. Second, a complete and unique root-to-sink path on the tree can be identified for each case in $D_{2}$. Third, assume we have the estimated posteriors from the past failure data before conduction of routine maintenance, and these are now used as priors to generate the data that would be observed after the routine maintenance.

Here, the prior independence assumptions are still assumed to be valid so that conjugate sampling can be characterised. To simulate from the intervened system instead of the idle system, the florets $F\left(w_{2}\right),F\left(w_{3}\right),F\left(w_{4}\right)$ are stochastically manipulated in response to the routine maintenance, and we adjust the corresponding Dirichlet hyperparameters as described in the previous section.

It is possible to embody the effects of this intervention when learning the causal structure by incorporating the stochastic manipulations we developed in the previous section into the MAP algorithm. We can check whether this improves the causal structure learning and parameter estimations. On the corresponding missingness event tree, see Figure 2, we accordingly revise the Dirichlet hyperparameters of florets $F\left(v_{1}\right),F\left(v_{5}\right),F\left(v_{6}\right)$ and $F(v_{7})$ using the method we proposed in Section 3.2.2.

We defined ϕ in Equation (27) to add uncertainties to the intervened floret distributions. In this study, we aim to compare the estimates learned from the best scoring model selected by the algorithm when no distributions are manipulated, i.e., ϕ=1, with the estimates learned from the best scoring model selected by the algorithm when inputting ϕ<1. In particular, we consider six different cases here: ϕ=0.1, ϕ=0.3, ϕ=0.5, ϕ=0.7, ϕ=0.9, and ϕ=1.

Now, we run the algorithm for α=0.001,α=0.01,α=0.1,α=1,α=3,α=5, where α is the prior parameter representing the number of phantom units entering the root node. We assess the resulted models in terms of situational errors [31] and MAP scores. The situational error (The total situational error of a tree is evaluated as $\gamma \left(T\right)=\sum_{v\in V_{T}}\left|\right|\theta_{v}^{*}-{\tilde{\theta }}_{v}{\left|\right|}_{2}$) for a situation *v* measures the Euclidean distance between the true conditional probabilities $\theta_{v}^{*}$ and the mean posterior probabilities ${\tilde{\theta }}_{v}$ estimated on the best scoring model.

**Figure 8:**
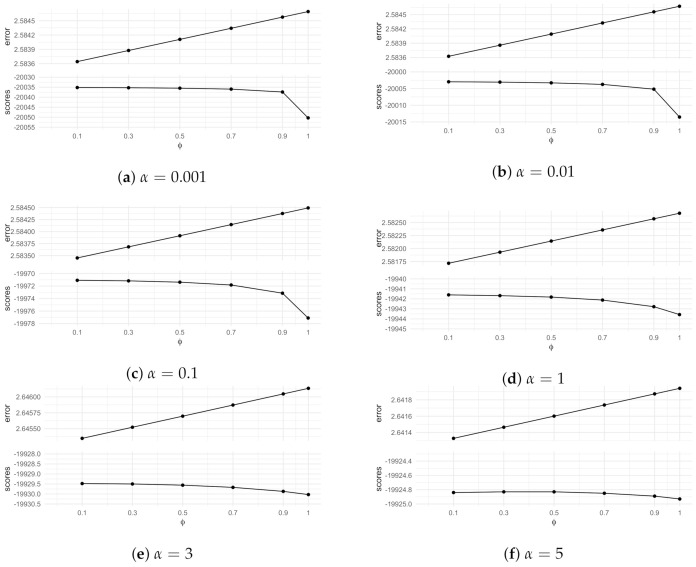
Comparing situational errors and MAP scores for the best scoring models selected to fit *D*_2_. The x-axis of each plot is labelled by different values of *ϕ*, where *ϕ* = 1 refers to the case when no manipulation is imported to the prior. Each plot displays results for a specified total phantom number α.

The results are shown in Figure 8. The upper panel of each plot displays the total situational errors, while the lower panel displays the MAP scores for the best scoring models for different values of ϕ. For any prior parameter α we choose, we observe that the best scoring model is selected from the algorithm by setting ϕ=0.1, which gives the smallest situational error and the highest MAP score. In particular, the situational error rises when ϕ increases towards 1. Thus, the posterior parameters are better estimated by incorporating the manipulations into the learning algorithm when modelling the experimental data for an intervened system.

When ϕ=1 (i.e., the distributions are not manipulated), the MAP score in each plot of Figure 8 is much lower than that for ϕ=0.1. This means the best structure selected with ϕ=0.1 is more consistent with the dataset $D_{2}$ than the best model selected by the algorithm without importing stochastic manipulations.

## 5. Discussion

Thus far, we demonstrated how the context-specific CEG is a compelling graphical tool for analysing system failure data. This happens not only because of its ability to represent structural asymmetries but also its flexibility in being able to perform the necessary analyses in a straightforward way even in the presence of censored data that are informedly missing; causal analyses can be performed through simple MAP structural learning algorithms. We developed bespoke causal algebras for the routine intervention and extended the back-door theorems for identifying its causal effects on the MCEG. The results from our designed experiments confirm the usefulness of these bespoke causal algebras in structural learning to improve the predictions needed for system reliability.

One concern of the study is that the model classes containing the best explanation can become huge when the systems are very large. However, the established methodology allows us to scale up the search space for more complex models with up to 20 variables [32]. Furthermore, these challenges associated with scalability are generic ones and are currently being actively researched. Each new development can be simply translated into causal analyses of reliability systems using the technologies we described above.

## Figures and Tables

**Table 1:** Mean posterior probabilities $P(X=1|stage,D_{1})$.

	$X_{leak}=1$			$X_{alarm}=1$		$B_{s/b}=1$		$X_{s/b}=1$			$X_{fail}=1$			
stage	$w_{2}$	$w_{3}$	$w_{4},w_{5}$	$w_{6},\cdots ,w_{9}$	$w_{10},\cdots ,w_{13}$	$w_{14},\cdots ,w_{19}$	$w_{20},\cdots ,w_{24}$	$w_{25},w_{26},w_{27}$	$w_{28},w_{29},w_{30}$	$w_{31},w_{32},w_{33}$	$w_{34}$	$w_{35}$	$w_{36}$	$w_{37}$
estimate	0.77	0.69	0.50	0.69	0.49	0.51	0.29	0.80	0.67	0.51	0.78	0.70	0.59	0.45

## Data Availability

The data used to support the findings of this study are available from the corresponding author upon request.
